# Restoring Susceptibility Induced MRI Signal Loss in Rat Brain at 9.4 T: A Step towards Whole Brain Functional Connectivity Imaging

**DOI:** 10.1371/journal.pone.0119450

**Published:** 2015-04-06

**Authors:** Rupeng Li, Xiping Liu, Jason W. Sidabras, Eric S. Paulson, Andrzej Jesmanowicz, Andrew S. Nencka, Anthony G. Hudetz, James S. Hyde

**Affiliations:** 1 Department of Biophysics, Medical College of Wisconsin, Milwaukee, Wisconsin, United States of America; 2 Department of Dermatology, Medical College of Wisconsin, Milwaukee, Wisconsin, United States of America; 3 Department of Radiation Oncology, Medical College of Wisconsin, Milwaukee, Wisconsin, United States of America; 4 Department of Anesthesiology, Medical College of Wisconsin, Milwaukee, Wisconsin, United States of America; Penn State University, UNITED STATES

## Abstract

The aural cavity magnetic susceptibility artifact leads to significant echo planar imaging (EPI) signal dropout in rat deep brain that limits acquisition of functional connectivity fcMRI data. In this study, we provide a method that recovers much of the EPI signal in deep brain. Needle puncture introduction of a liquid-phase fluorocarbon into the middle ear allows acquisition of rat fcMRI data without signal dropout. We demonstrate that with seeds chosen from previously unavailable areas, including the amygdala and the insular cortex, we are able to acquire large scale networks, including the limbic system. This tool allows EPI-based neuroscience and pharmaceutical research in rat brain using fcMRI that was previously not feasible.

## Introduction

Resting-state functional connectivity MRI (fcMRI) has been extensively studied using echo planar imaging (EPI) based methods [1]. Functional networks in humans [1–3] have been discovered that involve both long range and local connectivity. Because of the anatomical and structural characteristics of animals in the aural region, especially rats, deep brain structure coverage in fcMRI remains deficient. EPI signal dropout is significant in the insular cortex, amygdala, and the hypothalamus. It has been suggested that EPI signal dropout is a result of magnetic susceptibility of air in the ear canal of the rat. As a result, only a small portion of these brain structures is available for fcMRI studies [4–9].

The magnetic susceptibility effect is composed of two types, variations of the resonance field that are intravoxel and that are intervoxel in and near the region of interest. Intravoxel effects are reduced by use of short echo times and small voxels, with the voxel dimension in the slice encoding direction most significant. Intravoxel dephasing results in signal loss that cannot be recovered. Intervoxel susceptibility effects result in image distortion, which can be corrected by producing a magnetic field map.

The rat ear can be divided into three parts: external ear, middle ear, and inner ear. The inner ear is composed of bony and membranous labyrinths and is filled with fluid. The susceptibility effect is likely not caused by the inner ear. The middle ear and the external ear are both filled with air. Together they form a two compartment structure that is separated by the tympanic membrane.

Air is paramagnetic because of the magnetic properties of the O_2_ molecule. It seems to be more troublesome than bone, and the effects of air–tissue interfaces do not seem to be ameliorated by intervening bone, although quantitative data are lacking. We have equipment in place that allows relative, but not absolute, measurements of the susceptibility of materials. The fluorocarbon Fomblin Y is nearly identical in these measurements to Teflon. In the experiments reported here, this liquid-phase fluorocarbon seems to match the magnetic susceptibility of brain tissue. It is introduced into the ear canal to displace air and to match the magnetic susceptibility of brain tissue.

In a previous study, toothpaste was used to fill the external ear canal in an attempt to correct this problem [10]. The EPI signal dropout caused by air in the external ear canal was reduced in a 2 Tesla MRI system [11]. With the development of hardware and technology at high field strength, fcMRI has become a major tool for brain connectivity studies. However, the signal dropout increases with field strength and is not corrected at 9.4 T with the application of toothpaste. When toothpaste is applied to the rat ear, it only fills the external ear canal. Thus, this procedure does not eliminate the large air pocket in the middle ear.

**Fig 1:**
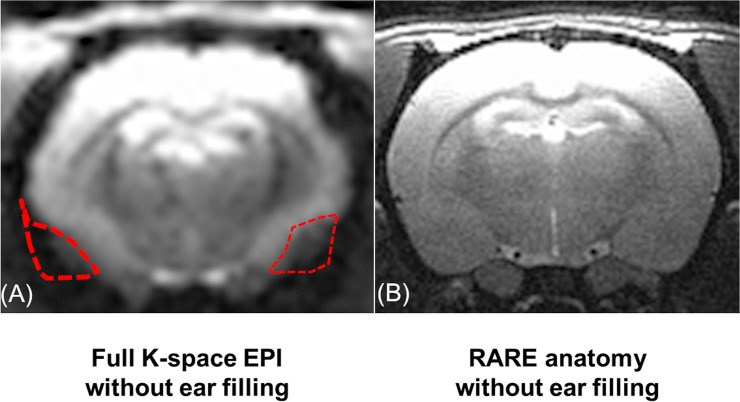
EPI and RARE comparison showing EPI signal dropout. (A) Comparison at 9.4 T of full k-space EPI in rat brain and (B) RARE anatomy. Regions of significant signal dropout (outlined in red) occur when gradient recalled EPI is used. Because of the use of a receive-only surface coil, image intensities, as acquired, vary from very bright to very dark, dorsal to ventral. They were leveled using gamma adjustment software. The same image processing algorithm was used in all MRI images of this paper.

Fig 1A is an example of an EPI image of a rat brain without the methods that are introduced in this paper. The slice thickness is 1 mm, and the in-plane resolution is 0.4 × 0.4 mm. Toothpaste has been applied to the external ear canals in this image. Compared to the rapid acquisition with relaxation enhancement (RARE) image of the same slice, Fig 1B, it is obvious that the majority of the insular cortex and almost the entire amygdala are affected by the susceptibility artifact. This makes fcMRI study of these two areas almost impossible. For example, there is no apparent possibility to use current shims to address the pointillistic character of the distortion of the homogeneity of the magnetic field near the middle ear canal of rat brain.

## Materials and Methods

Animals were anesthetized under isoflurane inhalation or dexmedetomidine infusion during the study. Animals were euthanized with saturated potassium chloride under isoflurane anesthesia after experiment.

The study protocol was reviewed and approved by the Institutional Animal Care and Use Committee (IACUC) of the Medical College of Wisconsin, AUA00001177. The Medical College of Wisconsin is a leader in the ethical and humane use of animals for research. MCW, through IACUC, takes responsibility for the humane care and use of laboratory animals. All protocols are reviewed by this committee, and investigators must comply with the "Principles for the Utilization and Care of Vertebrate Animals", the provisions of the Animal Welfare Act, and other applicable laws and regulations.

A tympanostomy tube, also known as an ear tube, is used in a clinical procedure to treat chronic otitis and recurrent acute otitis. After treatment, the tympanic membrane heals within a short period of time. The application of this surgical procedure in the rat followed by injection of a liquid-phase fluorocarbon into the middle ear demonstrates that the signal dropout caused by the magnetic susceptibility of air in the ear can be removed. It is hypothesized that this technique could be applied in survival animal models since the liquid can be easily removed from the ear canal after scanning [7].

Five male Sprague-Dawley (SD rat, Charles River Laboratories, Wilmington, MA) rats weighing between 300–450 g were used in this study. The left ear drum was penetrated and Fomblin Y (Sigma-Aldrich) was injected into the middle and external ear of all rats. We call this procedure “middle ear Fomblin filling,” MEFF. The right external ear canals of these five rats were filled either with gel toothpaste or Fomblin Y, as indicated, and the tympanic membranes were kept intact. All rats were given free access to food and water and were kept in a home cage with 12 hours of day/night light alternation for at least one week before the experiment.

General surgical protocol: All protocols and procedures were approved and carried out under the guidance of the MCW Institutional Animal Care and Use Committee. Dexmedetomidine hydrochloride (DexDomitor, Zoetis, KALAMAZOO, MI) has been used successfully in fcMRI studies and was used here [13,14]. The rat was placed prone, and anesthesia was provided by 2% isoflurane vaporized into 30–70% O_2_/N_2_ during surgery. Isoflurane was tapered off during the fcMRI portion of the study, and a continuous subcutaneous infusion of DexDomitor (0.1 mg/kg/hr) was used for maintenance of sedation. The rat was placed on a heated bench and supplied with 30–70% O_2_/N_2_. During fcMRI acquisition, pulse oximetry (8600V, Nonin Medical, Plymouth, MN), temperature, respiration rate, and inspired/expired O_2_ and CO_2_ (POET IQ2, Criticare Systems, Waukesha, WI) were monitored (WinDaq Pro, DataQ Instruments, Akron, OH) and maintained within normal physiologic ranges.

**Fig 2:**
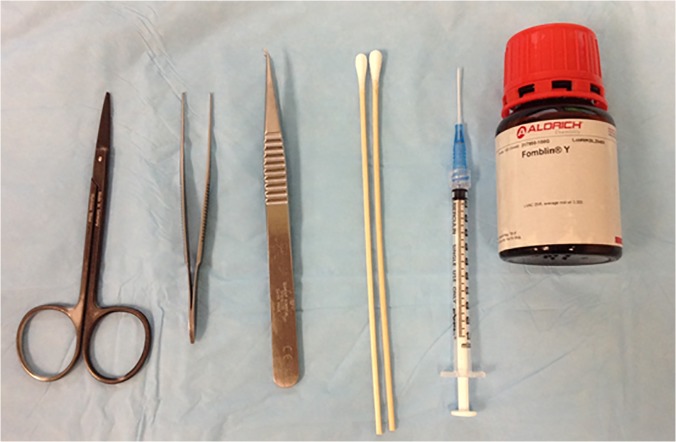
Instruments and materials needed for the MEFF procedure. A 23 gauge silicon needle was used. Fomblin Y with molecular weight of 3,300 g/mol has viscosity that is suitable for this procedure.

Fig 2 shows the equipment and fluorocarbon needed for this procedure. A 23-gauge silicon needle was used for the tympanostomy. The silicon needle was inserted into the external ear canal to about 10 mm deep. When it broke through the tympanic membrane, a clear, cracking sound was heard. The silicon needle was then inserted a further 5 mm to reach the end of the middle ear. Following the silicon needle placement, Fomblin Y, with a molecular weight of 3,300 g/mol, was injected to fill the air pocket in both the middle ear and the external ear. This compound has been widely used in MRI studies, especially diffusion tensor imaging (DTI) since it is proton-free and chemically inert [15]. For comparative purposes, we only filled the left ear of each animal in this study. Once the ear canal was filled with Fomblin Y, a plug made with medical grade cotton soaked in Fomblin Y was placed inside the external ear canal to seal the space and keep the fluid from leaking out. About 0.2 ml of Fomblin Y was used to fill the ear canal. Positive caloric reflex [16] can be observed by the end of the procedure, indicating a successfully filled ear. After the procedure, the rat was placed on a customized holder and sent for fcMRI studies.

### MRI Image Acquisition

Each rat was placed on a custom-designed cradle fabricated with G-10 fiberglass material, which has a magnetic susceptibility similar to air. A 9.4 Tesla MRI system with a 31 cm horizontal bore (Biospec Avance 94/31; Bruker, Karlsruhe, Germany) was used for scanning. A Bruker linear transmit-coil (T10325) was used with the axis of the coil coincident with the B_0_ axis and approximately with the cephalo-caudal axis of the rat brain. The central X-Y plane of the transmit coil was 3 mm in front of the ear canals. Signal acquisition was achieved using our self-designed 10 mm receive coil with low-noise-amplifier (LNA) displaced about 2 cm from the RF coil. The center of this receive coil was centered in the transmit coil. The coupling circuit uses an American Technical Ceramics (Huntington Station, NY) 800R series, high-Q, non-magnetic capacitor, a Rogers RT/duroid 5880 low-loss circuit board with 1-ounce copper trace on both sides, and a WanTCom (Chanhassen, MN) WMA9RA LNA, with an input impedance of 1.5 Ohms and an overall gain of 28 dB.

A combined symmetrical RT-shim and self-shielded gradient system (B-GA 12S RT) was used to improve magnetic field homogeneity. High order shimming was performed before scans. In order to conform to resolution levels used in most research, three sets of parameters were used. (1) Full k-space, single-shot gradient-echo EPI. *TE* = 18.76 ms, *TR* = 2 s, matrix size 96 × 96, FOV = 3.84 cm, BW = 300 kHz, 20 overscan lines, number of repetitions = 110, 10 contiguous interleaved 1 mm slices. (2) half k-space *TE* = 10.78 ms, *TR* = 2 s, matrix size 96 × 96, FOV = 3.84 cm, BW = 300 kHz, 20 overscan lines, number of repetitions = 110, 10 contiguous interleaved 1 mm slices. And (3), parameters used in our previous study [7] to achieve 200 micron cubic voxels: half k-space *TE* = 10.78 ms, *TR* = 2 s, matrix 128 x 128, BW = 400 kHz, slice thickness = 200 microns. The third set of parameters was used only for high resolution EPI. A RARE anatomical image was acquired before EPI scans with a 256 × 256 matrix, *TE* = 12.5 ms, *TR* = 2.5 s, and the same slice geometry as the EPI sequence.

### Data Analysis

Data processing was performed using analysis of functional neuroimages (AFNI) software [17]. Motion correction was performed for all individual scans before any further analysis (AFNI, 3dvolreg). Datasets were detrended to eliminate linear drifts, and separate slices were aligned to the same temporal origin. All resting-state acquisitions were analyzed individually using seed-voxel-based fcMRI analysis after they were registered to the same anatomical template (FSL, FLIRT). The seed region was chosen from the left insular cortex, consisting of 26 voxels, and the left amygdala, consisting of 16 voxels, across two slices. These seeds cannot be found on the right side of the cortex where an ear tube procedure was not performed. A band-pass filter was used for all resting-state EPI acquisitions with a low-pass filter of 0.1 Hz and a high-pass filter of 0.01 Hz on a voxel-by-voxel basis covering the entire brain. The resting-state, voxel-wise correlation coefficients were analyzed using the reference time course of each individual scan. Full k-space images were smoothed with 0.3 mm FWHM and underwent Fisher-Z transformation. The *p*-value for fcMRI results was set at 0.05.

## Results

**Fig 3:**
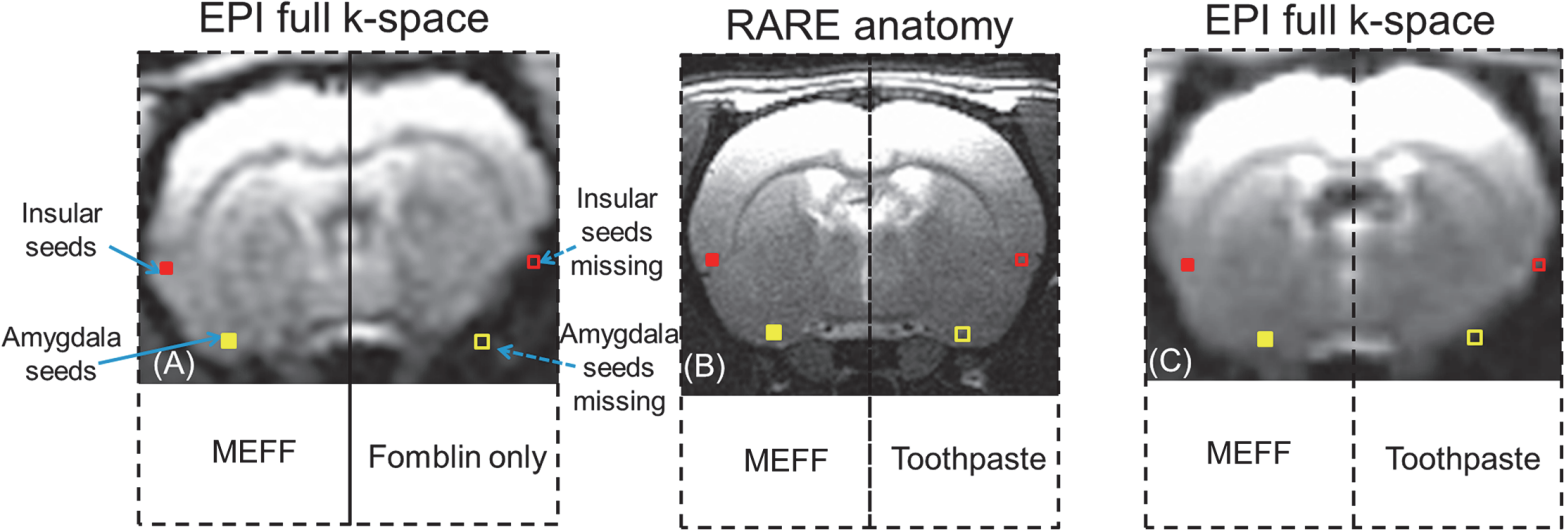
Comparison of EPI signal dropout with and without the MEFF procedure. A full k-space sequence was used, and all parameters were held constant. (A) Comparison of the use of MEFF, left, and of Fomblin in the external ear, right. MEFF restores the EPI signal in deep brain areas, and the result is highly comparable to the RARE image (B). Filled squares define seed regions used later for fcMRI analysis (red for insular cortex seeds and yellow for amygdala seeds). Open squares represent the seed regions that are lost because of magnetic susceptibility dropout. (C) Comparison of MEFF, left, with toothpaste in the external ear, right.

Fig 3 shows the results of EPI imaging with the same parameters as in Fig 1. It is clear that the EPI signals in the insular cortex and the amygdala were restored on the left side of brain. However, on the right side, where the MEFF procedure was not performed, EPI signal dropout remains significant. Fig 3A demonstrates the significance of the ear tube procedure. Although Fomblin Y was filled into both external ears of the rat, EPI signal was only restored in the left hemisphere, which shows that the air pocket in the middle ear is the major source of the magnetic susceptibility effect in the deep brain area. The effect cannot be removed by filling the external ear canal alone. The newly acquired EPI image on the left side of the brain is comparable to the RARE image (Fig 3B).

Toothpaste has been used as a material to fill the external ear canal in an attempt to reduce the susceptibility artifact. Fig 3C shows a comparison of MEFF on the left and external ear toothpaste filling on the right. The right sides of Fig 3A and Fig 3C show a comparison of toothpaste with Fomblin that is only in the external ear. Effects are similar. The EPI signal dropout remains significant compared to the nearly complete EPI coverage on the ear tube side. The effect of the MEFF procedure is significant in many slices that cover a large portion of the brain. In Fig 3A we also show the seed selection from the insular cortex and amygdala areas that are used in later analysis. In the left hemisphere, it is clear that seeds from the insular cortex (solid red square) and the amygdala (solid yellow square) were recovered by the restored EPI signal. In the right hemisphere, both of these seeds are outside of the clear EPI coverage (red and yellow squares).

**Fig 4:**
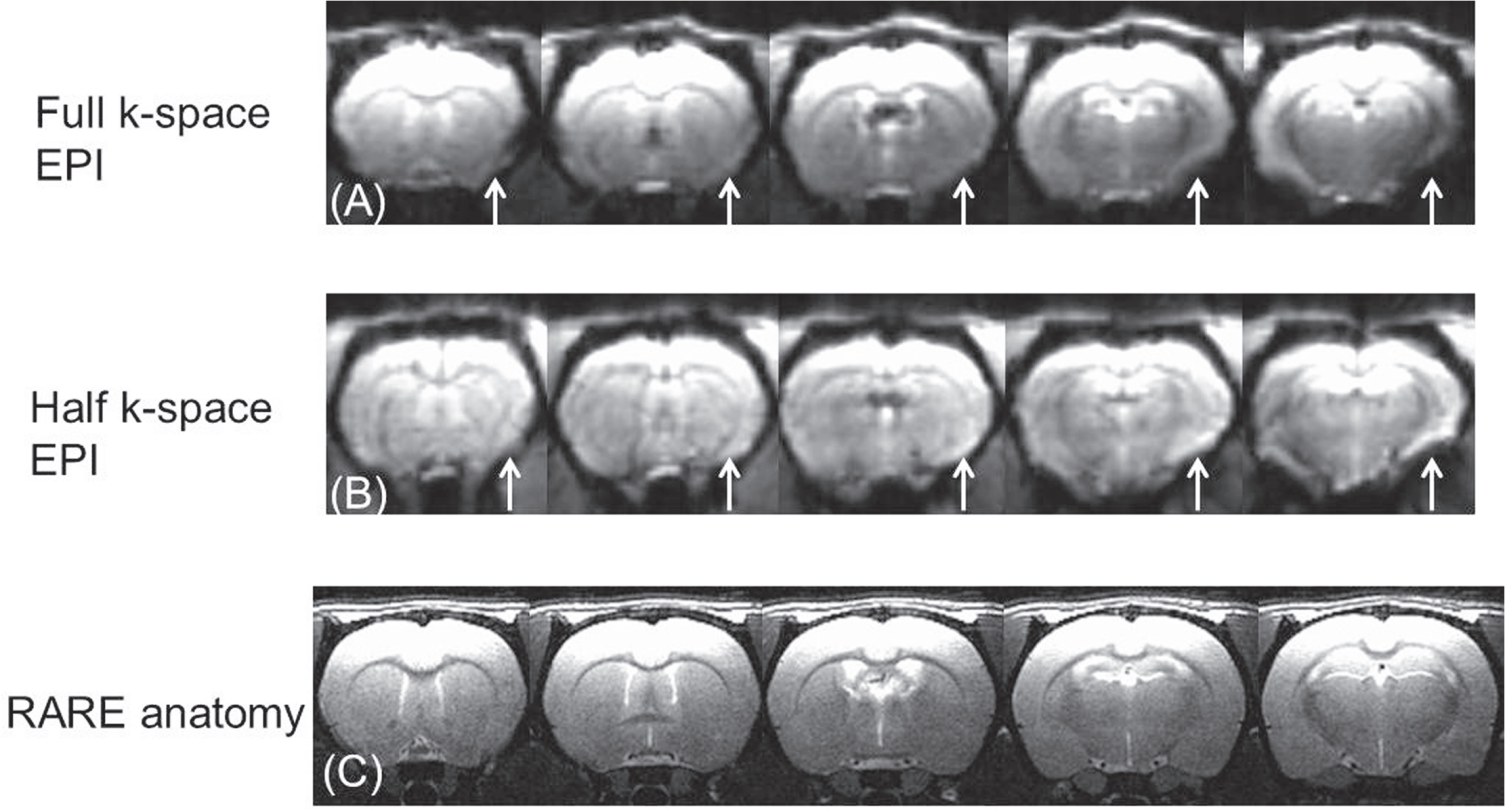
Comparison of EPI signal loss between full k-space and half k-space EPI scans in five contiguous 1 mm slices. MEFF was applied to the left side. White arrows point to the EPI signal dropout on the toothpaste-filled side. (A), (B) Full and half k-space EPI, respectively. (C) Rare anatomy.

A possible alternative way to reduce the EPI signal loss in the deep brain area is the use of the partial k-space sequence to reduce the echo time. Fig 4 demonstrates the EPI results in five consecutive 1 mm slices. We used our self-designed 1 cm diameter surface coil with a local LNA. It is clear from Fig 4A (right brain hemisphere) that despite the slight improvement in EPI signal loss in the deep brain area, many deep brain structures are still missing in the EPI image without the MEFF procedure compared to the anatomical image (Fig 4C). In the left hemisphere, this signal loss is recovered in regions of brain that are close to the ear canal when the MEFF procedure is used, and the insular cortex and amygdala areas can be easily identified. It is noted that the signal dropout in the most inferior slice of Fig 4 remains as an unsolved problem.

**Fig 5:**
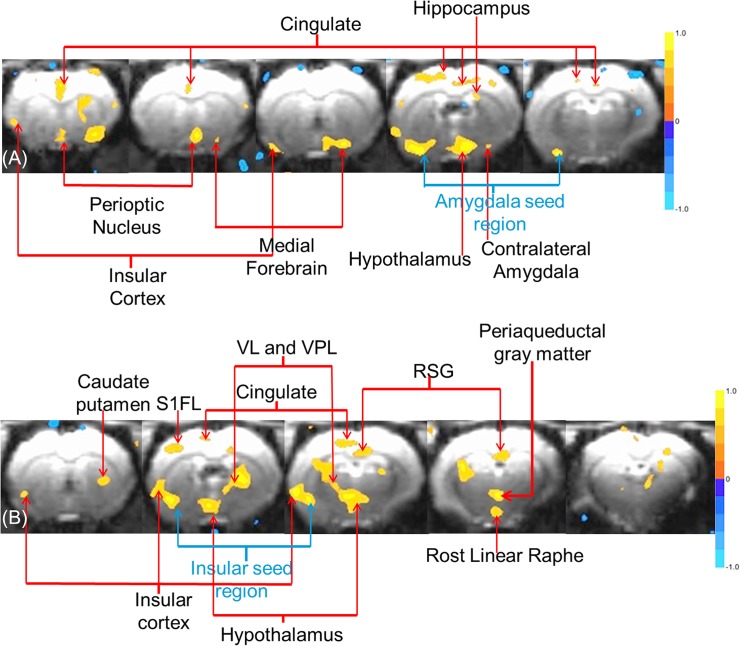
Resting state fcMRI using seed from deep brain areas. (A) fcMRI network using regionally averaged seeds from the amygdala and (B) the insular cortex.

We have demonstrated that we are able to eliminate much of the EPI signal loss in the deep brain, especially around the insular cortex and amygdala areas. It is interesting to demonstrate the application of this technique in an actual neuroscience study. Fig 5A was acquired using two seed regions from the left amygdala, one as indicated in Fig 3 and the other in an adjacent plane. The first set of imaging parameters was used, see the MRI Image Acquisition section. These parameters are unfavorable from the point of view of intravoxel dephasing. Nevertheless, good data were acquired when using Fomblin. Due to the incomplete coverage of the EPI signal on the non-Fomblin-filled side (right side), we were only able to acquire a small portion of the right side amygdala and insular cortex with fcMRI analysis. However, we can acquire a functional network containing the hypothalamus, insular cortex, hippocampus, and cingulate cortex using amygdala seeds. The bilateral sensory-motor cortex is also involved in this network. Fig 5B was acquired using insular cortex seeds from the left side. fcMRI analysis reveals a complex network of insular cortex, caudate putamen, cingulate cortex, retrosplenial granular cortex (RSG), ventral lateral thalamus (VL), ventral posterolateral nucleus (VPL), periaqueductal gray matter, rost linear raphe (Rli), and basal forebrain.

## Discussion

Elimination of signal dropout that arises from intravoxel dephasing in rat fcMRI studies is essential for neuroscience research. Due to the anatomical properties of the rat skull, EPI signal drop-out in deep brain areas is very common and affects many rat fMRI and fcMRI studies. EPI signal loss in this area is widely hypothesized to be a susceptibility artifact that is caused by the air–bone–brain tissue interface [10,18]. With the MEFF procedure, we are able to eliminate the air compartment inside the middle ear.

Fomblin Y is a proton-free liquid material that has been widely used in diffusion tensor imaging. It is inert with no known hazards or toxicity [19]. The magnetic susceptibility of this material is very close to Teflon. These properties make Fomblin Y a good choice as a filling material to remove the susceptibility artifact of the air in the middle and external ear canals. As shown in Fig 3A, we are able to restore the lost EPI signal in the insular cortex, hypothalamus, and amygdala areas compared to the EPI side (right hemisphere). The EPI image of the left hemisphere is comparable to the RARE image (Fig 3B). Simply filling the external ear canal with toothpaste or Fomblin Y does not have a significant effect on the EPI signal loss in deep brain areas. This study also confirms the previous hypothesis that the susceptibility of the middle ear is the main cause of the artifact in rat EPI of the deep brain area. The inert property of Fomblin Y and its liquid form make it a suitable choice for survival animal models. Due to the fact that Fomblin Y is a proton free and ion free material, it is possible that it can be further modified by adding paramagnetic metal ions. We may be able to create a solution with susceptibility that closely matches the surrounding tissue.

A desirable first step in use of the MEFF method is to shim the brain as well as possible using available current shims. The commercial Bruker current shims in the scanner used for the work presented here are of high quality and are adjusted automatically. More advanced shim strategies are described in the research literature [12, 20, 21]. A promising technique for the reduction of intravoxel dephasing is the use of multi-shot partial k-space echo-planar imaging (EPI) for high-resolution fMRI [22]. This method could be useful in reduction of signal dropout that was observed in the inferior slice of Fig 4. Signal loss is observed at the surface of the cortex images presented here. The use of an agar gel cap has been found to reduce this artifact in mouse brain [23]. In addition, since loss of signal intensity is mostly governed by the slice thickness, it follows that the thinnest possible slice should be used.

**Fig 6:**
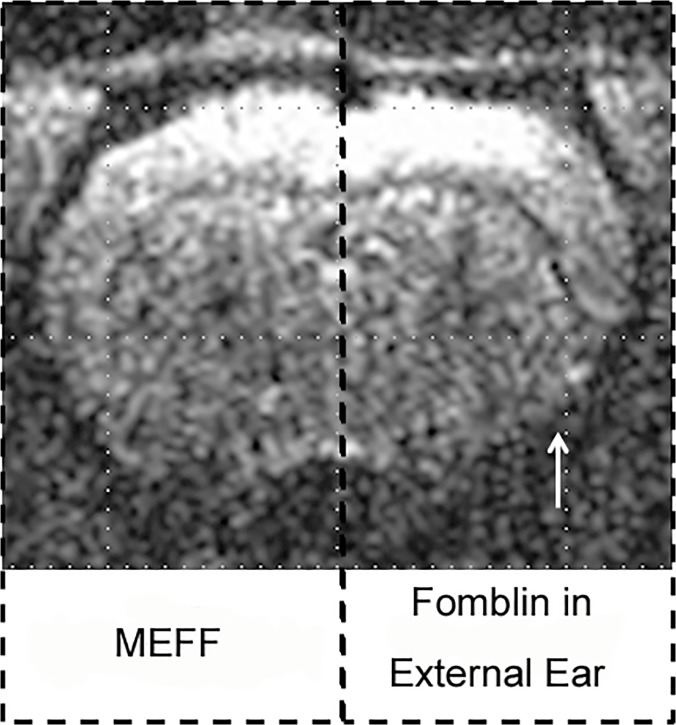
EPI image at 200 micron slice thickness (200 micron cubic) resolution showing that the magnetic susceptibility signal dropout still occurs and can be eliminated by MEFF. Significant EPI signal dropout (white arrow) in the insular cortex and amygdala areas is seen in the right side. An additional observation can be made: signal dropout in the dorsal-lateral regions is seen when 1 mm slices are used but not when 200 micron slice thickness is used. It is presumed that this particular signal dropout is due to close proximity to the ocular and nasal voids. It is significantly reduced by use of thin slices. Compare this image with others in this paper.

Researchers have tried many ways to minimize the ear canal susceptibility artifact, including filling with toothpaste [10], reducing slice thickness [14], and reducing *TE* by applying the partial k-space sequence [14,22,24,25]. The results of these methods are less than ideal for restoration of the signal. Minimizing the susceptibility artifact by reducing the slice thickness is effective in many cases, but also can reduce MRI signal intensity. Reduction of the echo time is effective but reduces BOLD contrast. With the help of our improved RF coil design, we are able to collect EPI data at 200 micron resolution in all three dimensions [14]. Even at this high resolution, significant loss of EPI signals in the deep brain areas occur, which can be avoided by use of the techniques of this paper. Fig 6 is an example of an EPI image at 200 micron cubic resolution. The MEFF procedure was done in the left ear, and the right ear was filled with Fomblin only. Methods and parameters remain the same as in our previous work [14]. These parameters were selected to minimize intravoxel dephasing. However, even at this high resolution, significant improvement in EPI coverage can be seen in the amygdala and insular cortex areas when Fomblin is used. The voxels in Fig 6 are twenty times smaller in volume than the voxels in other images of this paper, and the resulting EPI signal is lower. The EPI parameters that were used to obtain Fig 5 are unfavorable for reduction or elimination of intravoxel dephasing. Nevertheless, use of Fomblin in the middle ear was sufficient to greatly reduce intravoxel dephasing. The EPI parameters that were used to obtain Fig 6 are very favorable for reduction or elimination of intravoxel dephasing, but they were insufficient, and Fomblin was found to be necessary. This use of Fomblin in the middle ear is found to be both necessary and sufficient over a wide range of conditions. All MEFF procedures were performed with Fomblin also in the outer ear, which may have provided some additional benefit.

This paper introduces the method of fcMRI for validation of strategies for improving the magnetic field homogeneity over the rat brain. Voxels in gray matter in brain are connected in various ways that can be as close as the adjacent voxel or as distant as the extent of the whole brain. Thus the study of fcMRI in deep brain regions provides both the motivation for the present study and the means to evaluate the success of the MEFF method in reduction of intravoxel dephasing.

Due to the difficulties in EPI coverage in deep brain areas, there are no publications that used fcMRI in the deep brain regions of Fig 5. The insular cortex, hypothalamus, and amygdala are among the most important functional structures. They work as hubs for long range functional networks, and data from these structures are crucial. The function of these structures has been widely studied in human connectome research since human skull anatomy is vastly different from that of the rat [26–30]. With the techniques demonstrated in this study, true whole brain coverage seems to be within reach for fcMRI research using rats.

In order to demonstrate the efficacy of our MEFF procedure, we performed fcMRI analysis with seeds chosen from both insular cortex and amygdala areas based on the brain atlas. As part of the limbic system, the amygdala plays an important role in memory, learning, and emotion. In this study, when seeds from the left amygdala were used for analysis (Figs 3 and 5A), we found an extensive fcMRI network of the bilateral sensory-motor cortex, hypothalamus, insular cortex, hippocampus, and cingulate cortex [31–39]. Similarly, the insular cortex has been widely studied as an important structure for awareness, motor control, and emotions. We also found a complex functional network that connected the insular cortex to the caudate putamen, cingulate cortex, RSG, VL, VPL, periaqueductal gray matter, nuclear raphe, and basal forebrain (Fig 5B) [40–51]. Although these connections have been found in previous studies, our study is the first to show them by functional connectivity based on cross-correlation of the fluctuations found in resting-state time courses. With the MEFF procedure, these networks and interactions become available for *in vivo* neuroscience and pharmaceutical research.

The amygdala has rich anatomical projections with widespread subcortical and cortical regions. Because BOLD functional connectivity does not differentiate between afferent and efferent connections, these are considered together here. Also, the amygdala consists of at least six larger nuclei that, for brevity, will not be distinguished here. The subcortical connections of the amygdala include those with the basal forebrain, such as the nucleus Basalis, substantia innominata, septal preoptic area and the hypothalamus; the thalamus, especially medial, mediodorsal, and ventral nuclei, the striatum including the caudate putamen and n. accumbens; and numerous nuclei of the brainstem, the largest of which is with the parabrachial nucleus. The cortical connections of the amygdala are mainly with visual, auditory and somatosensory sensory association, polysensory cortices, the insula, the medial and orbitofrontal (limbic) frontal regions, and the hippocampus. In the work described here, connectivity from the amygdala seed region was revealed in the insular cortex, perioptic nucleus, medial forebrain, cingulate and hippocampus as indicated by red arrows in Fig 5A.

The insular cortex is a functionally heterogeneous region with subregions that integrate gustatory, visceral, vestibular, and nociceptive/thermosensitive sensory inputs together with somatic and limbic information. Accordingly, the cortical afferent connections of the insula are widespread, including entrorhinal, perirhinal, orbitofrontal, and cingulate projections, gustatory, olfactory and vestibular cortical inputs, as well as primary and higher-order somatosensory and auditory association fibers. The insula receives thalamic projections from ventromedial and various ventral posterior nuclei and the amygdala [52]. Connectivity from the insular seed region was observed to the caudate putamen, cerebrum, cingulate, ventral lateral and ventral posterior lateral regions of the thalamus, retrosplenial granular cortex, linear raphe, and periaqueductal regions. Connectivity was also observed to other insular regions. See Fig 5B.

This study provides a basic tool for rat fcMRI studies. With it, EPI coverage can be extended to deep brain structures. However, magnetic susceptibility effects in other regions of the brain may require other procedures for reduction or elimination, Fig 4.
